# Controversies and challenges of vaccination: an interview with Elizabeth Miller

**DOI:** 10.1186/s12916-015-0508-z

**Published:** 2015-10-16

**Authors:** Elizabeth Miller

**Affiliations:** Public Health England, 61 Colindale Ave, London, NW9 5EQ UK

## Abstract

Although strong evidence exists that the benefits of vaccination by far outweigh potential adverse events, controversy still exists. This has led opponents of vaccination to question its safety, efficacy and necessity. In an interview with Professor Elizabeth Miller, we discuss the continuum of beliefs held by vaccine refusers and hesitators, the resulting health consequences, and ways in which health professionals and industry regulators can help promote transparency to better convey the substantial health benefits of vaccination.

## Introduction

**Fig. 1:**
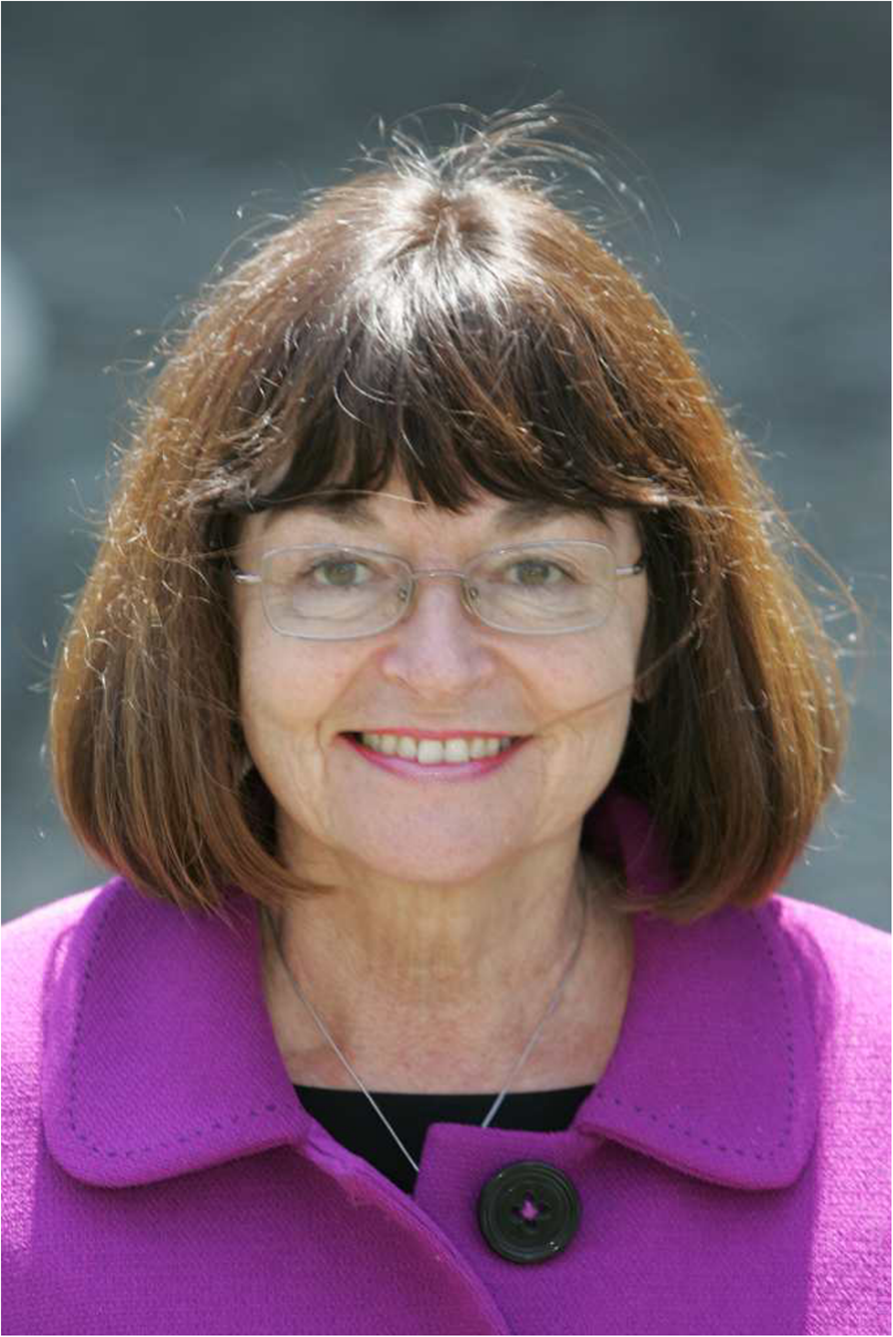
Professor Elizabeth Miller

Professor Miller (Fig. 1) is a Consultant Epidemiologist at the Immunisation Hepatitis and Blood Safety Department, Public Health England (PHE) in Colindale North, West London, and has a long standing interest in the risks and benefits of vaccination programmes. She leads a research team that undertakes trials of new vaccines or new schedules for existing vaccines and has been involved with trials of acellular pertussis, measles, mumps and rubella (MMR), *Haemophilus influenzae* type B (Hib), meningococcal C vaccines and, more recently, the pneumococcal and HPV vaccines. In collaboration with colleagues at PHE, she has conducted several vaccine safety studies to investigate the associations between MMR and autism, convulsions and “immune overload”, H1N1 vaccine and narcolepsy, thiomersal and developmental delay, oral polio vaccine and intussusception, and meningococcal C conjugate vaccine and nephrotic syndrome, among others. She was one of the founder members of the World Health Organization (WHO) Global Advisory Committee on Vaccine Safety and served for 6 years as a member of the WHO Strategic Advisory Group of Experts.

### 1) As a world-leading expert in immunization research you have become known as a key advisor on vaccination policies. Can you explain what attracted you to this area of research?

I first became involved in vaccine research in 1978, when I joined the then Public Health Laboratory Service as a medical epidemiologist. I worked on the large post-licensure safety and efficacy studies of pertussis vaccines that were being conducted following the collapse of the UK whooping cough immunisation programme in the mid-1970s. This massive decline in vaccine coverage was the result of allegations that the vaccine caused brain damage based on reports of children who developed neurological conditions after vaccination. These safety concerns were amplified by claims that the vaccine was also largely ineffective in protecting against the disease. Massive nationwide epidemics of whooping cough followed the collapse in coverage and, while it was relatively easy to demonstrate the efficacy of the vaccine, safety studies were more difficult to perform. Back then, before the advent of desk top computers and the internet, it was quite a challenge to conduct large epidemiological studies to test whether a temporal association between vaccination and the development of a rare clinical condition was due to chance or was evidence of a causal connection. It therefore took several years to conduct the prospective cohort and case control studies that eventually confirmed the safety of the vaccine and allowed coverage to be restored.

The next vaccine safety scare to hit the UK occurred in the 1990s, when claims were made that MMR caused autism. By this time, it was possible to remotely access computerized health records and link these with immunization records, which meant that retrospective cohort studies could be rapidly undertaken with minimal cost. Over the last two decades, our research team has conducted many studies of vaccine safety using these large linked databases and employing a novel statistical method that we developed to improve analytic efficiency and minimize confounding – a potential source of bias in any observational study of association. I have found this vaccine safety research not only intellectually challenging but also very rewarding in that its results can directly inform vaccine policy and the risk-benefit evaluation implicit in every individual’s decision to accept a vaccine.

### 2) What are the controversies regarding vaccines?

Vaccines differ from other medications as they are given to millions of healthy individuals, usually children, to prevent diseases that may no longer pose an immediate threat. Concern about vaccine safety is therefore perfectly legitimate and, when potential safety signals arise, they must be investigated promptly and rigorously. Many of the concerns about vaccine side effects have arisen as a result of reports of a temporal association between administration of a vaccine and development of a rare disease for which the cause is currently unknown. Such case reports are still the way that most safety signals are generated; indeed, health professionals and parents are encouraged to report any side effects that they suspect may have been caused by a vaccine to authorities such as the Centers for Disease Control and Prevention (CDC) in the US or the Medicines and Healthcare products Regulatory Agency (MHRA) in the UK.

In other instances, signals are generated from ecological associations, where an increase in the incidence of a disease is noticed to coincide with introduction of a vaccine. Examples of vaccine safety concerns that have arisen in this way are the alleged association between the combined MMR vaccine and autism, which was based on both a reported temporal link for some cases and a suggested ecologic association. This developed into a “controversy”, as despite sound epidemiological studies showing no excess of autism onsets after MMR vaccine, there remained a vocal lobby that did not accept the evidence.

The reasons why scientific evidence on vaccine safety is rejected by the so-called ‘anti-vaxxers’ are complex and varied. They include distrust of the scientific ‘establishment’ who generally promotes vaccination, albeit based on careful risk-benefit analyses, suspicion that those conducting vaccine safety studies are in the pay of the pharmaceutical industry, sincerely held personal belief that their child was damaged by a vaccine, or pseudoscientific beliefs such that vaccination is unnatural and it is better for a child to experience the real disease or that giving several vaccines to a young child can ‘overload the immune system’. Despite strong scientific arguments against the immune overload hypothesis and epidemiological studies showing no increased risk of infections after multiple vaccinations, this idea may seem plausible to parents who then hesitate to vaccinate their infant believing that deferral until an older age may be in their best interests. The concept of vaccine hesitancy is one that is now gaining currency as it recognizes that there is a continuum between those who accept and those who refuse vaccination and that it is not helpful to characterize all of the latter as anti-vaxxers.

### 3) What are the health consequences of refusing vaccinations and what are the implications beyond those who do so?

An implacable rejection of vaccination, despite strong evidence of its safety, is damaging. It places the individual at increased risk of disease and also of exposing vulnerable contacts. For example, in a measles outbreak in San Diego in which the index case was an ‘intentionally unvaccinated’ child who contracted the disease while travelling abroad, 75 % of the secondary cases were similarly unvaccinated due to refusal, with one hospitalization of an infected infant who was too young to be vaccinated. In addition to the clinical consequences of such outbreaks, there are economic impacts because of the extensive public health measures that need to be put in place to limit transmission and protect vulnerable contacts. However, the consequences can be even more far reaching than this, as the promulgation of anti-vaccine views via websites and the media can lead to a decline in vaccine acceptance among others who are not, in principle, opposed to vaccination. In the case of the MMR controversy, for example, sustained interest in the alleged association by the British media with extensive coverage of the unsubstantiated claims of the anti-vaccine lobby and scant attention to the actual scientific evidence resulted in a critical fall in MMR vaccine coverage in the UK. As a result, measles, which is highly infectious and can exploit even a small decline in population immunity, returned with savage consequences. After an interval of 14 years with no acute measles deaths, the resurgence associated with MMR vaccine refusal led to the deaths of two immunocompromised children who could not be vaccinated and therefore contracted the disease. Within the European region, refusal or hesitancy to accept the MMR vaccine because of unfounded safety concerns has been one of the factors behind its continuing failure to attain the region’s measles elimination goal, initially set for 2010, then deferred until 2015.

### 4) How should the risks of not vaccinating be communicated?

There is no blanket approach to communicating with parents who refuse or are hesitant to vaccinate. In fact, some research has suggested that emphasizing the dangers of not vaccinating may be counterproductive. In a controlled trial in the US, parents were randomly allocated to a control group or to receive 1 of 4 interventions, namely (1) information about the lack of evidence that MMR causes autism from the CDC, (2) information about the dangers of the diseases prevented by MMR, (3) images of children with diseases prevented by the MMR vaccine, and (4) a dramatic narrative about an infant who almost died of measles. None of the interventions increased parents’ intent to vaccinate. Furthermore, amongst those with the most negative opinions, the interventions actually increased misperceptions or reduced vaccination intention. Since parents rate their children’s doctor as their most trusted source of vaccine safety information, it is important that paediatricians and general practitioners have access to the most up-to-date information. In the UK, for example, during the MMR/autism controversy, the Department of Health posted each new safety study on its website as it was published, together with up-to-date information on disease risks.

Tailoring the message to the individual is important as well. In an attempt to provide health professionals with a framework for communicating with parents, researchers have identified five distinct parental attitudes, representing a continuum of beliefs regarding vaccination. There are the ‘unquestioning acceptors’ (30–40 %), who believe in the benefits of vaccination and trust their healthcare provider to have their child’s best interests at heart, and the ‘cautious acceptor’ (25–35 %), who have some minor concerns but nevertheless proceed with vaccination following a brief discussion on side effects and disease risks. The ‘hesitant’ group (20–30 %) comprises those who have significant concerns about vaccine risks. For this group, trust in their doctor or nurse is key and, if their questions are answered satisfactorily and completely by knowledgeable health professionals, they will proceed with vaccination. The ‘late or selective vaccinator’ (2–27 %) group has specific concerns about one vaccine or alleged phenomena, such as immune overload, and are generally knowledgeable. This group needs the most time, with detailed information on the risks and benefits of vaccination and, if necessary, another appointment to reconsider their decision. The outright ‘refuser’ of all vaccines generally comprise less than <2 % and are often motivated by their religious, philosophical or alternative beliefs. For this group, even referral to a specialist immunization clinic where they can have dedicated time with experts is unlikely to change their attitude. This analytic framework supports the concept of vaccine hesitancy and makes useful distinctions between the tiny minority who are implacably opposed to vaccination and the broader group of vaccine refusers who can have their views modified with appropriate discussion and information.

### 5) Should vaccination be compulsory?

Making vaccination compulsory by law is often seen as a way of improving compliance and dealing with the problem of vaccine hesitancy or refusal. While individual freedom of choice is an important principle, those who refuse vaccination not only pose a risk to themselves but also to others. The philosopher JS Mill, while a staunch proponent of individual liberty, recognized in his essay *On Liberty* in 1859, that “*the only purpose for which power can be rightfully exercised over any member of a civilized community, against his will, is to prevent harm to others*”, thus presciently providing an ethical justification for mandatory vaccination. Moreover, vaccine refusers are now often seen as ‘free loaders’ – exercising their rights to refuse vaccination safe in the knowledge that the risk to their own child is low because of the herd immunity provided by others who have their child vaccinated. In the European Union, 14 of the 27 countries have one or more mandatory vaccination programmes, with the legal consequences of failure to comply ranging from punitive measures such as pecuniary penalties, difficulty to attend public schools or even penal consequences for the parents, to milder disincentives such as just necessitating a formal ‘opt-out’ on religious or philosophical grounds. The rigour with which mandatory vaccination is enforced also varies and, overall, there is no clear evidence from European countries that mandatory vaccination necessarily achieves high coverage. In the US and Australia, however, compulsory vaccination has contributed to the success of immunisation programmes, but the balance between individual freedoms and public benefits is a fine judgement and not all countries will make the same decision. In countries such as Sweden, Norway, Denmark, the Netherlands, and the UK, compulsory immunisation is unlikely to be acceptable and indeed high coverage has been achieved through other approaches.

### 6) What can be done to increase transparency in research funded by pharmaceutical companies, and is enough being done to ensure that data from vaccine trials are being adequately reported, particularly regarding any adverse outcomes?

There have been significant changes to the regulatory framework in recent years which have ensured that manufacturers of any medicinal product, including vaccines, operate in a more transparent manner, with improved systems for detection of adverse events. Firstly, all those conducting clinical trials of vaccines and therapeutic drugs are encouraged to register them on a publically accessible website such as ClinicalTrials.gov. In the US, the Food and Drug Administration Amendment Act requires this by law and the International Committee of Medical Journal Editors (ICMJE) makes trial registration a condition of the publication of clinical trial results. The purpose is partly to ensure that potentially eligible participants are made aware of trials that may be of interest to them and are kept informed of the results, but also to reduce bias in outcome reporting. When an unexpected but serious adverse event occurs that might possibly be related to the vaccine or drug (termed a suspected unexpected serious adverse reaction), there is now a requirement to notify the licensing authority within 7 days so that information can be rapidly collated across different trials using the same product to assess whether this constitutes a safety signal. Hiding unfavourable safety results in a trial is not an option as the licensing authority can audit the conduct of the trial and the compliance of the investigators with Good Clinical Practice and the regulatory framework. As part of the licensing process, manufactures must also submit a Risk Management Plan that identifies possible risks of their product, including any potential pre-licensure safety signals, together with a mitigation strategy to reduce the risk. Thus, when the second generation of rotavirus vaccines was licensed, manufacturers were required to conduct large post-marketing studies to assess the risk of intussusception, as this rare side effect was the cause of the withdrawal of the first licensed rotavirus vaccine. With novel statistical methods, such as the self-controlled case series, and the increasing availability of large linked databases, such studies can now be conducted rapidly and with minimal bias.

Despite these improvements, no risk management plan could anticipate a rare and completely unexpected reaction, for example, narcolepsy following the AS03 adjuvanted H1N1 pandemic influenza vaccine, which first came to light as a result of observant sleep physicians who noted an increase in referral of cases, many of whom had recently been vaccinated. While pre-licensure trials were necessarily limited in size due to the urgency of getting the pandemic vaccine to the population in time, with a risk as low as 1 in 55,000 largely concentrated in children and adolescents, conducting a pre-licensure trial large enough to detect such a reaction would not have been feasible.

While it is important to convey the huge benefits of vaccination, it is also necessary to honestly acknowledge that the absolute safety of a new vaccine can only be confirmed after it has been given to hundreds of thousands of individuals and that the decision to vaccinate is a risk-benefit judgement that requires full informed consent.

### 7) Where can I find out more?

See reference list [1–16].
